# Elderspeak from the perspective of hospitalised geriatric patients—insights from a cross-sectional study using a mixed-methods design

**DOI:** 10.3389/fmed.2026.1893792

**Published:** 2026-07-29

**Authors:** Celina Friedland, Aline Schönenberg, Mia Lintl, Tino Prell

**Affiliations:** 1 Department of Geriatrics, Halle University Hospital, Halle (Saale), Germany; 2 Department of Geriatrics, Jena University Hospital, Jena, Germany

**Keywords:** ageing stereotypes, ageism, communication, elderspeak, older adults, patient centered, patient perspective

## Abstract

**Background:**

Elderspeak as a form of ageism is a simplified and patronising communication style characterised by diminutives, collective pronouns and exaggerated prosody. In healthcare, elderspeak can affect the physical and mental health of older patients. Understanding the patient’s perspective is essential for developing respectful and dignified communication strategies in contact with older patients.

**Methods:**

Video vignettes were used to analyse *N* = 45 geriatric patients’ perception of elderspeak compared to neutral language in a mixed-methods design. Quantitative data were analysed descriptively and using Wilcoxon test and mixed models, qualitative data were categorised inductively by Mayring.

**Results:**

All patients recognised elderspeak; 88.9% prefer neutral language over elderspeak. Elderspeak appears less professional and polite; qualitative responses indicate that elderspeak goes against normative communication, lacks respect and feels less warm or personal. In addition to linguistic form, contextual factors such as the interpersonal relationship also appear to be important for preferred communication.

**Conclusion:**

Geriatric patients recognize elderspeak as an unprofessional an non-normative form of communication that is widely disliked, although contextual factors and personal preference play a role. Overall, communication with patient is an important tool to foster appropriate health behavior and well-being. Future research and practice should aim to reduce elderspeak to avoid potentially negative effects on the well-being and health of older people.

## 1 Introduction

When interacting with older adults, a simple, infantilizing way of speaking is often used, sometimes unconsciously, which is known as elderspeak. This form of communication is characterized by the use of diminutives (“my dear”), collective pronouns (“we will wash”), simplified syntax and exaggerated prosody (loud, slow, overemphasized speech) (1). Elderspeak is sometimes based on implicit negative age stereotypes that reduce older people to deficit-oriented characteristics such as being in need of help or having cognitive limitations (2, 3). This stereotypical communication is an expression of ageism, which includes both conscious and unconscious ageism (4). The use of elderspeak can be motivated not only by age-related stereotypes, but also by the perception of cognitive and functional deficits sein (5, 6). Elderspeak can therefore be understood in the context of Communication Accommodation Theory, which describes how speakers adapt their linguistic communication to their communication partner (7). Although elderspeak is often well-intentioned (1), this style of language is associated with negative psychosocial consequences: it can reduce self-esteem (8), promote feelings of patronization (9, 10) and increase resistance in dementia care (11, 12). Nurses who use elderspeak are rated by observers as less respectful, competent and caring. In addition, patronizing language is often perceived as condescending and unfriendly (13–15). Existing literature has often focused on the views of naive observers such as younger adults, students, or caregivers (15, 16), while the perspective of older people themselves, particularly hospitalized patients, has been less included in previous elderspeak research. Another methodological disadvantage of earlier studies is that elderspeak was often only presented in written vignettes, without a realistic demonstration of spoken language (1). In view of demographic change, it is becoming more relevant to focus research on age-discriminatory language among hospitalized patients. In addition, there are only a few German-language studies on the subject so far (5, 6). The aim of this mixed methods study is to analyze age-discriminatory language from the perspective of older patients using video-based presentations of elderspeak and neutral language. Based on the current state of research, we formulate three hypotheses for the study:

H1: Older hospitalized patients recognize a linguistic difference between elderspeak and neutral language in the videos.

H2: The majority of patients prefer neutral language to elderspeak. Individual factors such as gender, educational level and mental well-being influence this preference.

H3: On a Likert scale for evaluating language styles, elderspeak is rated more negatively by older patients than neutral language.

In addition, a qualitative analysis will be used to collect individual reasons for each preference, as well as thoughts and feelings, evaluations of the way of speaking and the perceived quality of the relationship in the videos – for both ways of speaking. Findings on older patients’ views on elderspeak could help to improve communication in healthcare in the future.

## 2 Methods

### 2.1 Study design

The exploratory cross-sectional study with a mixed-methods design was conducted at the Department of Geriatrics at Halle University Hospital between May and July 2025. Inclusion criteria were: age >70 years, hospitalization as part of early rehabilitative geriatric complex treatment under OPS 8–550. Exclusion criteria were cognitive deficits relevant to everyday life (Mini-Mental Status Test (MMST) score <25), hearing and vision limitations, language barriers, dementia diagnosis, delirium or other conditions that restricted the ability to give consent. All subjects gave their written consent. The local ethics committee of Halle University Hospital gave a positive vote (number: 2025-051). All appropriate patients (purposive sampling) were contacted by the first author in their patient rooms (*n* = 50). The subjects were not explicitly informed about Elderspeak, but only that the study was analyzing communicative aspects of patient care to avoid bias. Five subjects rejected participation due to tiredness or visits from family members.

### 2.2 Sample size and sampling

The sample size was determined using a pragmatic mixed-methods design. For the qualitative part of the study, data saturation was used as a criterion, i.e., recruitment was stopped as soon as no new relevant topics were found in the participants’ open-ended responses. This limit was reached after interviewing 45 patients (17). The sample size also allowed us to do descriptive and inferential statistical analyses of the Likert scales with appropriate variance estimation.

### 2.3 Videos

The video sequences included each an identical everyday care situation between a patient and the nursing staff in neutral language and elderspeak. In the videos shown, only the communication style of the nursing staff changed. All patients watched both, the video in neutral language and the video with elderspeak. In the elderspeak video, we showed different characteristics of age-discriminatory communication in three different scenes: (a) diminutives (“my dear”) and informal address, (b) collective pronouns (“Have we taken our tablets yet?”) and (c) exaggerated prosody (loud, slow, overemphasized speech). To avoid carryover bias, the order of the two videos was randomly varied so that some patients saw the elderspeak video first and then the neutral video, while others were given the reverse order (see Appendices 1 and 2 for video development and video script).

Using a semi-structured questionnaire (see Appendices 3 and 4 for the questionnaire and questionnaire development), the subjects were asked to evaluate the two speech styles in the video sequences.

### 2.4 Covariates

In addition, the following data were collected to characterize the cohort: age, gender, marital status (single, married, divorced/widowed/separated), living situation (living alone, with partner/family, in a nursing home), level of education (low, middle, high), level of care, availability of care services and number of aids required in everyday life (glasses/visual aids, hearing aid, speech computer, walking stick, crutches, forearm crutches, rollator, wheelchair, other). In addition, geriatric assessments were extracted from patient files to describe patients in terms of cognition [MMST (18)], independence in everyday life [Barthel Index (19)], mobility according to the Tinetti test [(20)], and mental well-being [Geriatric Depression Scale (GDS) (21)].

### 2.5 Statistical analysis

Quantitative data were analyzed using the Statistical Package for the Social Sciences (SPSS Version 28.0.0) and R (Version 4.5.1). Categorical variables were presented as absolute frequencies or percentages. For metric variables, the mean, standard deviation and median as well as the 25% and 75% quantiles (interquartile range) were calculated. The significance level was set at 0.05 for all analyses and the tests were applied bilaterally. The Kolmogorov–Smirnov test was used to test for normal distribution. Due to significant deviations from normal distribution, Wilcoxon signed-rank tests were used for the Likert items. Effect sizes were reported as rank-biserial correlation (*r*) to evaluate the practical relevance of the differences. The Bonferroni-Holm correction was applied to control for multiple testing.

In addition, a cumulative link mixed model (CLMM) (22) was calculated to statistically account for the ordinal response data (1–5) and the dependence of the measurements within patients. The model included the condition (Elderspeak vs. neutral) as a fixed effect and a random intercept for the participants (for methodological details, see Appendix 5). In addition, a linear mixed model (LMM) was performed as a robustness test with the numerical response scale (1–5). For both models, estimate coefficients, standard errors, odds ratios or effect sizes, and 95% confidence intervals were reported. Due to the small sample size, the CLMM results should be interpreted exploratively; their trend and size were used to check consistency with the Wilcoxon results.

A qualitative content analysis according to Mayring (inductive categorization) was performed to analyze the qualitative data (23). The patients’ responses were coded by the first author and then classified into predefined categories by three independent raters using a codebook (see Appendix 6). All categories are reported in absolute frequencies with the aim of supporting the quantitative data. To determine the frequencies of the categories, a response was always assigned to a specific category (and thus counted once) if at least two of the three raters had classified that response in the same category.

In the event of disagreement between all three raters (i.e., no agreement between at least two), the response was not taken into account. Inter-rater reliability was determined using Fleiss kappa. The analysis was performed separately for each of the open-ended questions.

### 2.6 Hypotheses

H1: To test whether the participants recognized linguistic differences between the two videos, they were asked after watching the videos whether they had noticed any differences (yes/no item). In addition, they were given an open response field in which to describe the differences they had noticed. The frequencies of the elderspeak characteristics mentioned (diminutives, informal address, collective pronouns, exaggerated prosody) were presented descriptively in percentages. It was expected that the participants would be able to identify and name the linguistic differences.

H2: Language preference was evaluated using a trichotomous item (video 1, video 2, no preference). Based on previous research, a preference for neutral language was expected. In addition, qualitative information on the reasons for the preference was evaluated in order to identify individual motivations. The influence of gender, educational level and mental well-being (GDS) on preference was to be analyzed using logistic regression analysis.

H3: To evaluate the perception of elderspeak in comparison to neutral language, patients rated both videos using eight identical 5-point Likert items (1 = ‘strongly disagree’ to 5 = ‘strongly agree’) on respect, dignity, self-determination, dependence, appreciation, equality, self-confidence and self-esteem. A better rating of neutral language was expected. After testing for normal distribution (Kolmogorov–Smirnov, Shapiro–Wilk), Wilcoxon signed-rank tests were used in cases of non-normality. In addition, a cumulative link mixed model (CLMM) was used to account for the ordinal dependent data. Due to the small sample size of patients preferring elderspeak or when selecting certain item levels, the CLMM results should be interpreted as exploratory, as the estimation of random effects in small samples is associated with increased uncertainty.

In addition, qualitative data on thoughts, feelings, and assessments of speech patterns and perceived relationship quality were collected for both videos to further support the quantitative data.

## 3 Results

### 3.1 Sample description

In total, N = 45 patients (60% female) aged 81.58 ± 5.52 years participated in the study (Table 1). The majority of patients had middle to high education, mostly unimpaired cognition according to the MMST, no depressive symptomology according to the GDS, and lived independently either alone or with partner.

**Table 1:** Descriptive statistics of the sample variables.

Variables	Mean (SD)	Median	Quantile (25%, 75%)
Age	81.58 (5.52)	82.00	77.0; 86.0
MMST	28.07 (1.71)	29.00	26.50, 30.0
Barthel	40.44 (15.81)	35.00	30.0; 47.50
GDS	2.20 (2.16)	2.00	0.50; 3.50
Tinetti	13.66 (5.00)	14.00	10.0; 17.0
Number of assistive devices	2.40 (1.07)	2.00	2.0; 3.0

Variable	Category	*N*	Percentage
Gender	Male	18	40
	Female	27	60
Marital status	Single	1	2.2
	Married	19	42.2
	Divorced/separated	25	55.5
Living situation	Alone	25	55.5
	With partner/family	19	42.2
	Nursing home	1	2.2
Education	Low	1	2.2
	Middle	32	71.1
	High	12	26.7
Care level	None	17	37.8
	1	3	6.7
	2	18	40.0
	3	6	13.3
	4	1	2.2
	5	0	0.0
Care provided by nursing service	Yes	19	42.2
	No	17	37.8
	No, but by family	9	20.0

GDS, geriatric depression scale, MMST, mini mental status test. Level of education: low = no graduation; medium = vocational training; high = university degree.

### 3.2 H1: recognition of elderspeak

According to H1, all 45 patients recognized a linguistic difference between the videos with neutral language and elderspeak. The elderspeak video showed three typical characteristics: (1) diminutives and informal address, (2) collective pronouns, and (3) exaggerated prosody. These were recognized by the participants with different frequencies: 91.1% recognized diminutives, 53.3% recognized informal address, 55.6% recognized collective pronouns, and all (100%) recognized exaggerated prosody.

### 3.3 H2: language preference

**Figure 1:**
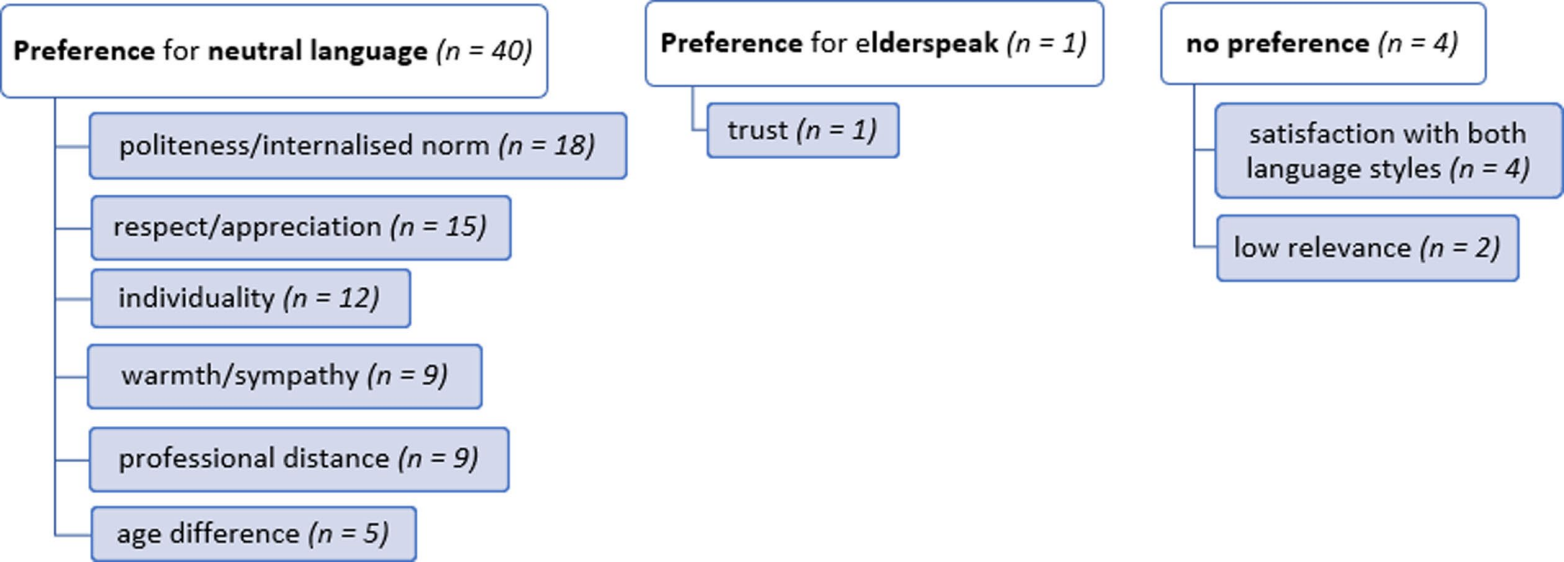
Category system for explaining the preference.

According to H2, 88.9% of patients favored neutral language, 2.2% favored elderspeak, and 8.9% did not indicate a preference. Open-ended responses were qualitatively evaluated to explain the reasons for their preferences (inductive content analysis; see Figure 1).

Six categories were identified in the reasons given for preferring neutral language (Figure 1). Politeness/internalized norm (*n* = 18) was mentioned most frequently: “It’s just the right way to talk” (P27). Respect/appreciation (*n* = 15) was represented by statements about respectful, dignified communication: “Because it was respectful and that’s how you interact with other people” (P33). Individuality (*n* = 12) referred to personal and individual address: “You are not seen as an object” (P17). Warmth/sympathy (*n* = 9) emphasized that neutral language seemed “nicer” (P6), “warmer and more sympathetic” (P4). The category professional distance (*n* = 9) referred to the desire for a factual, not too familiar form of address: “They are still a stranger, so I do not want to be addressed informally.” (P14). Finally, five participants mentioned the age difference as a reason: “I am at an age where I want to be addressed formally.” (P13).

One patient preferred elderspeak and explained that with trust: “It makes me feel comfortable, I trust the nurse.” (P15). Four other participants showed no preference and explained that they were satisfied with both styles of language (*n* = 4) or that it was not very relevant (*n* = 2): “I do not really care. I think both are good.” (P21); “In the end, it’s the help and the success of the treatment that matters, not the language.” (P29). Interrater reliability was *κ* = 0.78, 95% CI [0.73, 0.82].

The influence of individual factors (gender, educational level, mental well-being) could not be examined due to the small number of people who preferred elderspeak (*n* = 1).

### 3.4 H3: quantitative data from the Likert items

To evaluate elderspeak and neutral language, all patients completed eight identical 5-point Likert items (1 = “strongly disagree” – 5 = “strongly agree”) on respect, dignity, self-determination, dependence, appreciation, equality, self-confidence and self-esteem.

**Figure 2:**
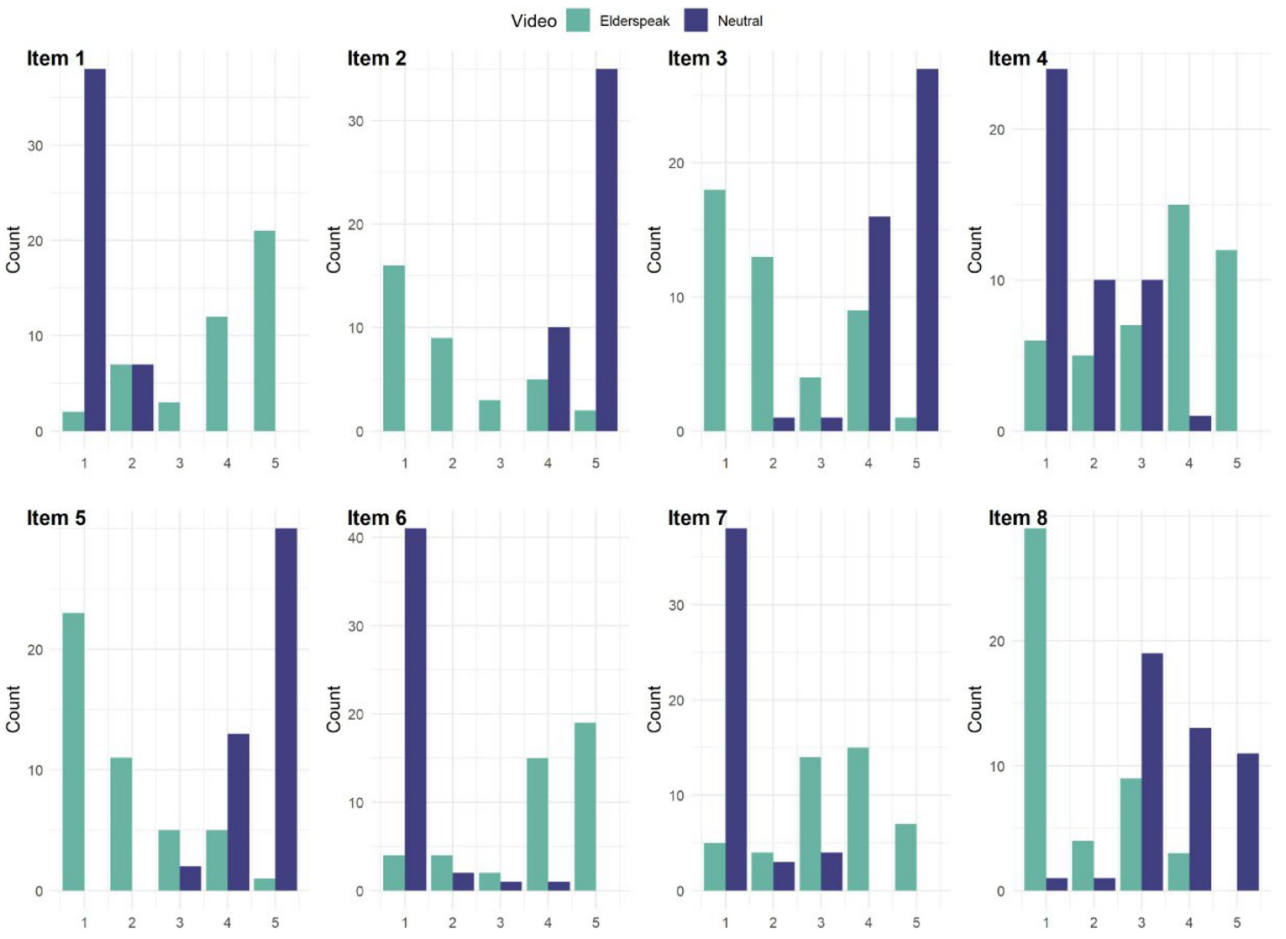
Illustration of absolute frequencies of Likert items: llustration for neutral language and elderspeak.

The descriptive analysis showed clear differences between elderspeak and neutral language for all items (Figure 2, Table 2). For example, item 1 (“I feel treated disrespectfully”) was rated M = 1.16 (SD = 0.37) in neutral language and M = 3.96 (SD = 1.26) in elderspeak. Tests for normal distribution (Kolmogorov–Smirnov, Shapiro–Wilk, Appendix 7) showed significant deviations, which is why non-parametric Wilcoxon signed-rank tests were used. Significant differences were found for all eight items (*p* < 0.001, Bonferroni–Holm corrected), with high effect sizes (*r* > 0.5) (Appendix 8).

**Table 2:** Descriptive description of items for neutral language versus elderspeak.

Item	Neutral		Elderspeak		Group comparison	
	M (SD)	MD (IQR)	M (SD)	MD (IQR)	*p*	*r*
1	1.16 (0.367)	1.00 (0.00)	3.96 (1.261)	4.00 (2.00)	<0.001	0.857
2	4.78 (0.420)	5.00 (0.00)	1.84 (1.224)	1.00 (1.00)	<0.001	−0.855
3	4.53 (0.661)	5.00 (1.00)	2.16 (1.224)	2.00 (2.00)	<0.001	−0.831
4	1.73 (0.889)	1.00 (1.50)	3.49 (1.359)	4.00 (2.50)	<0.001	0.699
5	4.62 (0.576)	5.00 (1.00)	1.89 (1.133)	1.00 (1.50)	<0.001	−0.843
6	1.16 (0.562)	1.00 (0.00)	3.91 (1.294)	4.00 (1.50)	<0.001	0.831
7	1.24 (0.609)	1.00 (0.00)	3.33 (1.187)	3.00 (1.00)	<0.001	0.811
8	3.71 (0.944)	4.00 (1.50)	1.69 (1.019)	1.00 (2.00)	<0.001	−0.775

Item 1: I feel that I am being treated disrespectfully as a patient. Item 2: I found the language used to be appropriate and dignified. Item 3: I feel that I can act independently and make my own decisions freely. Item 4: I feel that I am dependent. Item 5: I feel valued by the nursing staff. Item 6: I feel that I am being talked down to as a patient. Item 7: I notice that my self-confidence is decreasing. Item 8: The way I was spoken to increased my self-esteem. Likert scale: 1: strongly disagree. 2: somewhat disagree. 3: neutral. 4: somewhat agree. 5: strongly agree. Group comparison using Wilcoxon signed-rank test rank-biserial correlation *r*.

In the supplementary cumulative link mixed model (CLMM), a negative but insignificant trend was observed for elderspeak (*β* = −0.305, SE = 0.173, *z* = −1.76, *p* = 0.078; OR ≈ 0.74), which means a around 26% lower probability of positive ratings. The linear mixed model (LMM) confirmed a significant effect (*β* = −0.274, SE = 0.086, *t* = −3.19, *p* < 0.01). The direction and size stayed the same after adjusting for age and gender. Because of the small sample size of patients preferring elderspeak or when selecting certain item levels, the CLMM estimates should be seen as exploratory, but they show consistent trends across all models. The results did not depend on the order of the video presentation (Appendix 9).

### 3.5 Perception of elderspeak

For qualitative insights into the perception of language styles, the responses to three open-ended questions (thoughts/feelings, evaluation of speech style, evaluation of the relationship) were analyzed and categorized by topic (Table 3). The analyses consistently showed that patients rated neutral language mostly positively (feeling good, respected, professional) and elderspeak significantly more negatively (infantilized, unwell, disrespectful).

**Table 3:** Thoughts and feelings in neutral compared to elderspeak language.

Language	Category *As a patient, I feel…*	*n*	Example quote	*κ* (95%–CI)
Neutral	Well/good	35	‘Actually, I would feel good about it.’ (P30)	0.80 (0.71–0.88)
	Respected	6	‘I find that respectful.’ (P11)	
	Treated personally	5	‘I would feel that the nurse was responding to my needs.’ (P24)	
	Cared for professionally	5	‘I feel that I was treated correctly as a patient; that was professional work of the nurse.’ (P8)	
Elderspeak	Infantilised/put down	34	‘I would feel like I was being treated like a child, as if I were no longer quite right in the head (…) Last week, a nurse addressed me as “my princess,” which was completely inappropriate.’ (P33)	0.79 (0.71–0.86)
	Uncomfortable/not well	28	‘That would annoy me, it’s unpleasant.’ (P9)	
	Treated impersonally	4	‘It’s impersonal.’ (P3)	
	Neutral/no feelings	4	‘It’s important to me that I get help, I do not feel anything special about it.’ (P29)	
	Not respected	3	‘It’s disrespectful, you do not do that.’ (P17)	
	Positive perception	2	‘I would feel good.’ (P37)	

**Table 4:** Evaluation of speech style in neutral compared to elderspeak language.

Language	Category *The way of speaking was.*	*n*	Example quote	*κ* (95%–CI)
Neutral	Normal/good	28	‘That was completely normal.’ (P10)	0.95 (0.87–0.99)
	Warm/friendly	19	‘The nurse was friendly.’ (P2)	
	Professional	8	‘It was clear and understandable.’ (P39)	
	Respectful	5	‘It was respectful, like how you treat an adult.’ (P33)	
	Personal	2	‘The nurse treated people as human beings.’ (P17)	
	Negative perception	2	‘It seemed a bit rehearsed to me.’ (P26)	
Elderspeak	Childish/exaggerated	13	‘The nurse talks as if she were talking to a child.’ (P26)	0.86 (0.80–0.92)
	Disrespectful	13	‘It’s disrespectful, as if the patient were not an equally valued human being.’ (P28)	
	Cold/inappropriate	11	‘I found it rather cold and inappropriate.’ (P24)	
	Not good	10	‘I did not like it at all.’ (P2)	
	Impersonal/too personal	6	‘Too sterile (.), because it was so impersonal.’ (P20) ‘Too familiar.’ (P22)	
	Positive perception	5	‘Not 100% to be recommended, but still good.’ (P31)	

The most common categories, exemplary citations and interrater reliabilities are shown in Tables 3–5.

**Table 5:** Perception of the relationship between patient and nursing staff when using neutral language compared to elderspeak.

Language style	Category *The relationship between the nurse and the patient was…*	*n*	Example quote	*κ* (95%–CI)
Neutral	Normal/good	27	‘It was a normal patient–nurse relationship.’ (P8)	0.81 (0.73–0.88)
	Professional	8	‘It was correct and objective.’ (P4)	
	Respectful	7	‘As it should be, it was respectful.’ (P35)	
	Negative perception	7	‘It was a bit sterile.’ (P20)	
	Warm/friendly	5	‘I did not find it cold, but friendly.’ (P3)	
	Personal	2	‘The patient was recognised as a person.’ (P39)	
Elderspeak	Not good	13	‘The relationship was not good.’ (P45)	0.94 (0.87–0.99)
	Disrespectful/hierarchical	10	‘The relationship is as if the patient is the worst. ‘(P28)	
	Cold/inappropriate	10	‘There was a coldness in the nurse’s language.’ (P2)	
	Childish/too familiar	7	‘The patient was addressed more like a good mate. I would not want to be treated like that.’ (P18)	
	Impersonal	6	‘Somehow impersonal.’ (P12)	
	Positive perception	5	‘It was actually good, I would say.’ (P37)	

**Figure 3:**
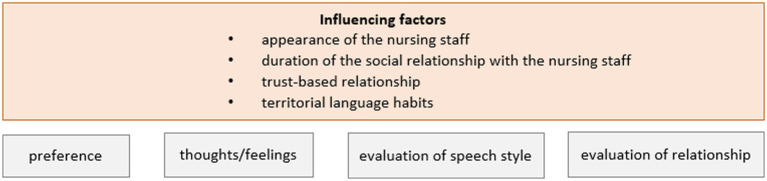
Factors influencing the perception of elderspeak.

In addition, the open-ended responses showed several factors influencing the perception of elderspeak (Figure 3). For many patients, the appearance of the caregiver was decisive: “It always depends on how the person approaches you.” (P29). The duration of the relationship also played a role: “If you have known the nurse for a long time, it’s different. Then she can say “my dear,” but not at the beginning.” (P43). The trust-based relationship was highlighted as a key factor: “You have to build trust first. If we do not know each other yet, I do not want to be addressed that way.” (P21). Finally, regional language habits also influenced the assessment: “In this region, people are often addressed as “my dear,” even outside the hospital.” (P8). Figure 3 summarizes the influencing factors that can modify the perception of elderspeak.

## 4 Discussion

Ageism shortens life expectancy, impairs physical and mental health, reduces health behavior and quality of life, and promotes social isolation (4). Elderspeak, as a linguistic expression of ageism, can therefore have direct negative consequences for older people. In an ageing population, it is therefore becoming increasingly important to reflect on ageist language. Since elderspeak occurs frequently, especially in nursing communication (24), it is essential to consider the patient’s perspective when developing preventive and educational interventions. Notably, elderspeak may lead to reactive behavior or even rejection of care, cumulating in stress and worse mental and physical health outcomes (12). Previous studies have mostly examined elderspeak from an observer’s perspective or in nursing home residents and people with dementia (11, 24, 25); empirical data from cognitively fit patients in acute geriatric care is currently missing. In addition, studies have often been based on written vignettes (10, 15), while studies using video-based auditory stimuli are rare. This study therefore closes a central research gap by examining patient-centred perceptions of ageist language in a realistic clinical context.

### 4.1 Recognition (H1)

As expected, all patients recognized clear linguistic differences between neutral speech and elderspeak. Diminutives and exaggerated prosody were identified more frequently than informal address or collective pronouns, which is probably due to their greater acoustic prominence in the videos. This shows that geriatric patients can clearly differentiate between different communication styles.

### 4.2 Preference (H2)

Almost 90% of participants preferred neutral language over elderspeak. This trend is consistent with previous studies, which also indicate that older people have a negative attitude towards elderspeak (26). The qualitative data provided to explain the preference for neutral language frequently showed themes of politeness, respect, individuality and warmth. The importance of respect and warmth in communication has already been mentioned in previous studies: older adults rated nurses who used elderspeak as less respectful (15) and warm-hearted (27). Due to the small number of people who preferred elderspeak (*n* = 1), individual influencing factors (gender, educational level, GDS) could not be analyzed; future studies with larger samples should examine this aspect. Nevertheless, the strong preference for neutral language illustrates the negative attitude of older patients towards elderspeak.

### 4.3 Evaluation (H3)

The quantitative analysis confirmed that elderspeak was rated significantly more negatively than neutral language. All eight Likert items showed significant differences in the Wilcoxon test (all *p* < 0.05, *r* > 0.5). Elderspeak was perceived as less respectful, dignified and appreciative, while at the same time stronger feelings of dependence and reduced self-esteem were reported. The CLMM showed a trend towards more negative ratings (*β* = −0.305, *p* = 0.078), and the LMM confirmed the effect (*β* ≈ −0.27, *p* < 0.01). Differences in significance can be explained methodologically, as ordinal models estimate more conservatively. However, the direction of the effects was the same in all analyses, suggesting that the results indicate a more negative perception of elderspeak compared to neutral language.

The qualitative results support these findings: elderspeak was described as infantile, demeaning, disrespectful, cold, and impersonal, while neutral language was associated with respect, warmth, professionalism, and personal address. The high interrater reliability (*κ* = 0.78–0.95) confirms the methodological reliability of the coding.

### 4.4 Influencing factors

Several participants pointed out that how elderspeak is perceived depends on the caregiver’s behavior, the level of trust, and the length of the relationship. Elderspeak was seen as less annoying when there was a close relationship – a finding that agrees with earlier studies in nursing homes (6, 9). Regional linguistic habits, such as forms of address like “My dear” (28), can also influence interpretation. Thus, it seems that not only the linguistic form, but also the relationship level and situational context are important for how elderspeak is perceived. Future studies should systematically record these influencing factors and examine the impact on the perception of elderspeak. This could lead to a more differentiated understanding of the conditions under which elderspeak is experienced as supportive or discriminatory. This is of high relevance, as the effect of elderspeak lies not only in momentary dissatisfaction: it cascades into stressful care situations or even rejection of care, poorer care quality, and thus worse health outcomes, highlighting the importance of proper communication not just for patient satisfaction, but for overall care quality and wellbeing (1, 12, 29).

### 4.5 Limitations

This study has several methodological limitations. Firstly, the sample size (*n* = 45) is small, which limits the statistical power of the CLMM analyses in particular, especially as the vast majority of patients preferred elderspeak, leading to unbalanced groups and small sample sizes for certain item levels of ordinal items. This limits the statistical power to detect small effects, and the reported *p*-values should be interpreted with caution. However, the consistent direction of the effects across different models suggests a robust trend. The resulting confidence intervals are wide, and the findings should primarily be interpreted as exploratory. Second, the study was conducted in a specific patient group – hospitalized older adults in German acute geriatric hospitals. This limits the generalization to other contexts, such as nursing homes, ambulatory settings or patients with cognitive impairments. Importantly, as this was an exploratory study to provide first insight into elderspeak from a patient perspective, which had not yet been done before, patients were required to fill out questionnaires and respond to the videos meaningfully. Therefore, patients with dementia or delirium were excluded from study participation. However, for interventions targeting elderspeak, it is crucial to include those patients, as they are oftentimes primary targets of elderspeak. Thirdly, elderspeak was presented in a standardized video-based vignette format specific to German culture; due to the exploratory nature of the study and the lack of German measurement instruments, the videos should also be considered exploratory. Although using videos allows for controlled comparability, the emotional impact of real interactions can only be reproduced limited. In addition, possible cultural influencing factors (e.g., regional language habits) were not systematically recorded. Future studies should capture these aspects in a differentiated way, include larger samples and analyze real nursing communication situations – for example, through video recordings or naturalistic observation designs. Overall, elderspeak in particular and communication in general is a highly impactful but complex topic in the care of older adults, reaching far beyond simple preference: elderspeak may be considered as a stressor for patients, cumulating in resistance to care and poor health outcomes. While the aim of our study was to provide first insight into the patient perspective on elderspeak in German hospital care, the cross-sectional design and lack of covariates cannot fully uncover the effect of elderspeak on older patients. Longitudinal and in-depth research needs to be performed to fully understand elderspeak and its impact on patients and care. Lastly, although the order of the videos was standardized, it cannot be ruled out that seeing a similar situation twice may have introduced a response bias. This was done to ensure situational comparability between the two conditions (elderspeak vs. neutral), meaning that any differences in video ratings were due to the communication style and not because of situational circumstances, conversational content or behavior. Still, a more naturalistic design taking a multitude of situations and persons into consideration may be more fruitful for future studies and interventional designs.

## 5 Conclusion

The results underline the need to increase awareness of ageist language in care settings and to specifically improve communication skills. Particularly in nursing training, training courses can be implemented to address this issue. Future research should focus on extending the presented results using longitudinal data under the inclusion of important covariates to understand the long-term effects of elderspeak on overall well-being and care quality. Intervention studies should be designed to reduce elderspeak in the long term in order to avoid negative effects on the well-being and health of older people.

## Data Availability

De-identified data will be made available upon reasonable request by the corresponding author.
