# Supplementary material for: Elderspeak from the perspective of hospitalised geriatric patients—insights from a cross-sectional study using a mixed-methods design
Source: Front Med (Lausanne). 2026 Jul 29;13:1893792. doi: 10.3389/fmed.2026.1893792 (PMC13461420; doi:10.3389/fmed.2026.1893792)
Supplement: Supplementary file 1 [file Table_1.DOCX]

Supplementary Material

# 1 Appendix 1: Video development

Various videos were designed and produced to examine elderspeak in comparison to neutral language (elderspeak vs. neutral language). The elderspeak language attributes shown were selected on the basis of current literature [18, 20]. Particular attention was paid to features that had been identified as particularly common in previous studies and those that could be clearly and realistically presented on video. In addition, a video was created using neutral language without elderspeak. The videos were produced independently with film technical support from the Dorothea Erxleben Learning Centre in Halle and a patient actor. The actress was informed in advance about the purpose and content of the recordings and gave her consent to the use of the video material in the study. All recordings showed identical content of an everyday clinical situation between an elderly patient and a nurse, who was played by one of the authors herself (CF). The simulation included a realistic welcome to the patient, medication check, washing and goodbye by the nurse. Due to the intimate nature of the washing process, this was only implied by washing one hand. The videos were produced to be comparable in terms of length, content and setting, but differed in the style of language used by the nurse towards the patient. The Elderspeak video showed three scenes with identical content that featured (1) diminutives (‘my dear’) and informal address, (2) collective pronouns (‘Are we all well today?’) and (3) exaggerated prosody (spoken slowly, loudly and with exaggerated emphasis). The video with neutral language showed the same scene without elderspeak. The videos were tested on members of the target group (n = 5) in a pre-test before the start of the study to check their understandability. The videos had an average duration of 1–2 minutes and presented the central content in a compact and easily understandable form. The video presentation was shown on the clinic's internal patient television system. During use on the ward, the videos were played with headphones to reduce external noise and make sure the spoken language could be understood clearly.

# 2 Appendix 2: Video script

## 2.1 Neutral vignette:

A patient is lying in bed early in the morning when a nurse enters the patient's room.

**Nurse**: ‘Good morning, Mrs. Müller. Are you feeling well?’

*The patient nods in agreement, with a slight smile.*

*The nurse looks into the medicine box and shakes it briefly. The box is already empty.*

**Nurse**: ‘Oh, Mrs Müller, you've already taken all your tablets for this morning.’

**Patient**: ‘Yes.’

**Nurse**: ‘Very good! Is it alright if I start with your personal hygiene now?’

*Patient nods in agreement.*

*The nurse puts on gloves and takes a washcloth from the washbowl.*

**Nurse**: ‘Right, Mrs Müller, I'll start by washing your arms. Please lift your left arm slightly.’

*The patient lifts her arm. The nurse begins the care and gently wipes the patient's left hand with the washcloth.*

- CUT -

*The personal care is complete.*

**Nurse**: ‘Right, Mrs Müller, that's it for today. If you need anything else or feel uncomfortable, please let me know.’

*The patient nods gratefully to the nurse.*

**Nurse**: ‘See you later, Mrs Müller.’

## 2.2 Elderspeak Vignette 1: Diminutives and informal address

*A patient is lying in bed early in the morning when a nurse enters the patient's room.*

Nurse: ‘Good morning, dear. Are you feeling well?’

*Patient nods in agreement.*

*The nurse looks in the medicine box and shakes it briefly. The box is already empty.*

Nurse: ‘Oh, my dear, you've already taken all your tablets for today.’

Patient: ‘Yes.’

Nurse: ‘That's great! Now I'll freshen you up, ok?’

*Patient nods in agreement.*

*The nurse puts on gloves and takes a washcloth from the washbowl.*

Nurse: ‘I'll start by washing your arms, my dear. Please lift your left arm slightly. What a good girl.’

*Patient lifts her arm. The nurse begins the care and gently wipes the patient's left hand with the washcloth*.

- CUT -

*The personal care is complete.*

Nurse: ‘There you go, dear, that's it for today. If you need any more help, just let me know, okay?’

*The patient nods gratefully to the nurse.*

Nurse: ‘See you later, dear.’

## 2.3 Elderspeak Vignette 2: Collective pronouns

A patient is lying in bed early in the morning when a nurse enters the patient's room.

Nurse: ‘Good morning. Are we feeling well today?’

The patient nods in agreement, with a slight smile.

The nurse looks into the medicine box and shakes it briefly. The box is already empty.

Nurse: "Oh, we've already taken all the tablets for this morning.

Patient: ‘Yes.’

Nurse: ‘Very good! Then let's freshen up now, ok?’

Patient nods in agreement.

The nurse puts on gloves and takes a washcloth from the washbowl.

Nurse: ‘Let's start by washing your arms, shall we? Please lift your left arm slightly.’

The patient lifts her arm. The nurse begins the care and gently wipes the patient's left hand with the washcloth.

- CUT -

The personal care is complete.

Nurse: ‘Right, that's everything done for today. If we need any help or anything else, we'll let you know, OK?’

The patient nods gratefully to the nurse.

Nurse: ‘See you later.’

## 2.4 Elderspeak Vignette 3: exaggerated prosody

Caregiver speaks exaggeratedly clearly, very loudly and deliberately slowly.

*A patient is lying in bed early in the morning when a nurse enters the patient's room.*

**Nurse**: ‘Good morning, Mrs. Müller. Are you feeling well?’

*The patient nods in agreement, with a slight smile.*

*The nurse looks into the medicine box and shakes it briefly. The box is already empty.*

**Nurse**: ‘Oh, Mrs Müller, you've already taken all your tablets for this morning.’

**Patient**: ‘Yes.’

**Nurse**: ‘Very good! Is it alright if I start with your personal hygiene now?’

*Patient nods in agreement.*

*The nurse puts on gloves and takes a washcloth from the washbowl.*

**Nurse**: ‘Right, Mrs Müller, I'll start by washing your arms. Please lift your left arm slightly.’

*The patient lifts her arm. The nurse begins the care and gently wipes the patient's left hand with the washcloth.*

- CUT -

*The personal care is complete.*

**Nurse**: ‘Right, Mrs Müller, that's it for today. If you need anything else or feel uncomfortable, please let me know.’

*The patient nods gratefully to the nurse.*

**Nurse**: ‘See you later, Mrs Müller.’

# 3 Appendix 3: Questionnaire

**Geriatrics Cover Sheet**

Version number: 2.0

Version date: 15 April 2025

Date:	_______________________

Patient code:	_______________________

Stay number:	________________________

Participation in studies:	________________________

________________________

________________________

________________________

________________________

| **GeriAddOn Routine assessment** | | | |
| --- | --- | --- | --- |
|  | **Assessment** | **available?** | **transfer** |
| Cognition | MoCA/MMST |  |  |
| Functionality | Barthel |  |  |
| Mobility | Tinetti |  |  |
|  | TuG |  |  |
| Depression | GDS |  |  |
| Social status | Nikolaus |  |  |
| **Elderspeak** | | | |
|  | **Questionnaire** | **available?** | **transfer** |
|  | Personal data |  |  |
|  | Perception of language |  |  |

**Questionnaire on personal data**

**Dear participant,**

Thank you for taking part in this study. The following questions are designed to help us learn more about you as a person. Please answer the following questions.

| 1 | How old are you? | ______ years |
| --- | --- | --- |
| 2 | What is your gender? |  male  female  diverse |
| 3 | What is your marital status? |  single  married   widowed / divorced / separated |
| 4 | What is your living situation? |  living alone   with partner/in a family   living in a nursing home   Others, please specify? _________________________ |
| 5 | What is your highest level of education?  (if school leaving certificate is mentioned, ask specifically about vocational training and studies) |  Primary school (8th grade)   Secondary school, commercial school, intermediate school leaving certificate (10th grade)   A-levels/vocational A-levels   Vocational training   Completed degree (university, technical college)   No qualification |
| 6 | Which aids do you use regularly in everyday life? |  Glasses/visual aids  Hearing aid  Speech computer   Walking stick  Crutches  Forearm crutches   Rollator  Wheelchair  Other: |
| 7 | What is your care level? |  none  1  2  3  4  5 |
| 8 | Are you cared for by a care service? |  Yes  No  No, but by relatives |

**Questionnaire on language perception**

**Dear participant,**

Thank you for taking part in this study. In the following questions, we would like to learn more about how you perceived the language used in the videos shown. There are no right or wrong answers. We are interested in your honest opinion. Please answer the following questions based on your perception of the two videos.

**Video 1**

1.1. First, we would like to learn more about your perception of the first video. The following questions refer **ONLY to the first video**. Please answer the open-ended questions in short sentences.

1.1.1 **How would you feel if someone spoke to you like this? Please describe your thoughts and feelings.**

……………………………………………………………………………………………………………………………………………………………………………………………………………………………………………………………………………………………………………………………………………………………………………………………………………………………………………………………………………………………………………………………………………………………………….

1.1.2 **How would you rate the nurse's way of speaking?**

………………………………………………………………………………………………………………………………………………………………………………………………………………………………………………………………………………………………………………………………………………………………………………………………………………………………………………………………………………………………………………………………………………………………………….

1.1.3 **How did you perceive the relationship between the nurse and the patient?**

………………………………………………………………………………………………………………………………………………………………………………………………………………………………………………………………………………………………………………………………………………………………………………………………………………………………………………………………………………………………………………………………………………………………………….

1.2. Imagine you are the patient in the video. Please answer to what level the following statements would apply to you. Mark the corresponding number on the scale. You can choose between the numerical value 1 for ‘I completely disagree’ and 5 for ‘I completely agree’. Again, all answers refer to the first video.

| 1) I feel that I am being treated disrespectfully as a patient. |  I completely  disagree  I completely  agree |
| --- | --- |
| 2) I found the language used to be appropriate and dignified. |  I completely  disagree  I completely  agree |
| 3) I feel that I can act independently and make my own decisions freely. |  I completely  disagree  I completely  agree |
| 4) I feel that I am dependent. |  I completely  disagree  I completely  agree |
| 5) I feel valued by the nursing staff. |  I completely  disagree  I completely  agree |
| 6) I feel that I am being talked down to as a patient. |  I completely  disagree  I completely  agree |
| 7) I notice that my self-confidence is decreasing. |  I completely  disagree  I completely  agree |
| 8) The way I was spoken to increased my self-esteem. |  I completely  disagree  I completely  agree |

**Video 2**

2.1. Next, we would like to learn more about your perception of the second video. The following questions refer **ONLY to the second video** and scenes 1, 2 and 3 contained therein. Please answer the open questions in short sentences.

2.1.1 **How would you feel if someone spoke to you like that? Please describe your thoughts and feelings.**

………………………………………………………………………………………………………………………………………………………………………………………………………………………………………………………………………………………………………………………………………………………………………………………………………………………………………………………………………………………………………………………………………………………………………….

2.1.2 **How would you rate the nurse´s way of speaking?**

………………………………………………………………………………………………………………………………………………………………………………………………………………………………………………………………………………………………………………………………………………………………………………………………………………………………………………………………………………………………………………………………………………………………………….

2.1.3 **How did you perceive the relationship between the nurse and the patient?**

………………………………………………………………………………………………………………………………………………………………………………………………………………………………………………………………………………………………………………………………………………………………………………………………………………………………………………………………………………………………………………………………………………………………………….

2.2. Imagine you are the patient in the video. Please answer to what level the following statements would apply to you. Mark the corresponding number on the scale. You can choose between the numerical value 1 for ‘I completely disagree’ and 5 for ‘I completely agree’. Again, all answers refer to the **second video with scenes 1, 2 and 3**.

| 1) I feel that I am being treated disrespectfully as a patient. |  I completely  disagree  I completely  agree |
| --- | --- |
| 2) I found the language used to be appropriate and dignified. |  I completely  disagree  I completely  agree |
| 3) I feel that I can act independently and make my own decisions freely. |  I completely  disagree  I completely  agree |
| 4) I feel that I am dependent. |  I completely  disagree  I completely  agree |
| 5) I feel valued by the nursing staff. |  I completely  disagree  I completely  agree |
| 6) I feel that I am being talked down to as a patient. |  I completely  disagree  I completely  agree |
| 7) I notice that my self-confidence is decreasing. |  I completely  disagree  I completely  agree |
| 8) The way I was spoken to increased my self-esteem. |  I completely  disagree  I completely  agree |

**Video 1 and video 2 in comparison**

3. Finally, we would like to ask you to compare the two videos shown. The following statements refer to **both videos**! Please answer the following questions in short sentences.

**3.1 Did you notice any differences between the first and second videos? If so, what differences did you notice in the language?**

- **Yes**
- **No**

………………………………………………………………………………………………………………………………………………………………………………………………………………………………………………………………………………………………………………………………………………………………………………………………………………………………………………………………………………………………………………………………………………………………………….

**3.2 Which way of speaking (video 1 or video 2) would you prefer to be addressed with? Please explain why.**

- **VIDEO 1**
- **VIDEO 2**
- **No preference**

………………………………………………………………………………………………………………………………………………………………………………………………………………………………………………………………………………………………………………………………………………………………………………………………………………………………………………………………………………………………………………………………………………………………………….

# 4 Appendix 4: Questionnaire development

The following demographic data were collected to describe the sample: age, gender, marital status, living situation (living alone, with partner, in a nursing home) and level of education. In addition, geriatric assessments were recorded to describe the patients in more detail in terms of cognition (MMST [1]), independence in everyday life (Barthel Index [12]), mobility (Tinetti [24]) and mental well-being (Geriatric Depression Scale (GDS) [29]). This data was routinely collected during hospitalisation and could therefore be taken from patient files. Data on the number of aids required in everyday life and information on the level of care and care services were also collected.

For each video (neutral and Elderspeak), we used identical open-ended questions to record the patients' thoughts and feelings, their assessment of the way of speaking, and their perception of the quality of the relationship in order to identify themes. To avoid suggestion and bias, open-ended questions were placed at the beginning of the questionnaire. In addition to the open-ended questions, eight Likert-type items were answered on a five-point scale (from 1: completely disagree to 5: completely agree). The items were created based on existing literature [3, 14] and included questions about the perception of respect, dignity, self-determination, dependence, appreciation, equality, self-confidence and self-esteem. These items were also identical for both videos in order to compare the responses between elderspeak and neutral language style. Finally, patients answered questions comparing the two videos: The perception of a difference between the videos was asked with a yes/no question. On top of that, the patients could say in an open-ended part what differences they noticed in detail to check the quality of the videos. Also, the preference for the language style was asked with a trichotomous single-choice question (preference for video 1, video 2, or no preference). Here, too, patients could additionally explain their preference with a free-text answer to support the quantitative data with qualitative data. The questionnaire was tested on a pilot sample (n = 5) to check how well it worked. Depending on what the patients could do, the answers to exploratory questions were partly noted down by the respondents themselves or by the interviewer (CF). The survey was conducted in the patient's room without hospital staff present. The interviewer remained neutral, avoided making suggestions and strictly adhered to the standardised questionnaire in order to increase the comparability of the responses. Care was taken not to evaluate or interpret the responses in order to minimise bias due to the interviewer's role. The survey took approximately 30-45 minutes.

# 5 Appendix 5: Methodological details of the CLMM analysis

Analyses were performed in R (4.5.1) version using the packages readxl, dplyr, tidyr, ordinal, lme4 and emmeans. Prior to modelling, we retained only the 16 pre-specified video-item variables and converted all item responses to the integers 1–5; any entries not interpretable as 1–5 were set to missing. Four negatively worded items were inverted so that, for every item, higher values uniformly indicate a more positive perception (greater perceived respect, self-confidence or self-esteem). Data were then reshaped to long format (one row per respondent × item × condition). Age was mean-centred for use as a covariate and sex was included as a categorical covariate where indicated.

The primary inferential approach used cumulative link mixed models (CLMM) for the ordered 1–5 responses with a random intercept for participant to account for within-subject dependence. The principal models estimated the effect of condition (elderspeak vs. neutral) using the formula response ~ condition + (1 | id) (ordinal logit link); models were fitted with adaptive Gauss–Hermite quadrature (nAGQ = 5). Secondary CLMMs included age and sex as fixed covariates (response ~ condition + age + sex + (1 | id)). For transparency and interpretability we report model coefficients (log-odds), corresponding odds ratios, and threshold estimates; estimated marginal responses and predicted probabilities were obtained using the emmeans package.

All CLMM results were complemented by a robustness analysis using linear mixed models (LMM) on the numeric 1–5 response scale (response_num ~ condition + (1 | id) and with covariates), fitted with lme4 (REML). Because sparse response cells can affect ordinal models, we inspected category frequencies and restricted item sets where appropriate; where necessary we report results after collapsing categories or omitting items with extremely sparse non-reference cells. Model diagnostics and both ordinal (CLMM) and quasi-metric (LMM) results are reported so that effect direction, practical magnitude (mean difference on the 1–5 scale) and ordinal inference are all transparently available.

# 6 Appendix 6: Codebook

Research question 1: How do patients explain their linguistic preferences?

**Categories for preference for neutral speech:**

| **Category** | **Definition** | **Coding rule** | **Example quotations** |
| --- | --- | --- | --- |
| Politeness/internalised norm | Statements that reflect formal politeness, social etiquette or internalised norms of appropriate behaviour. | Inclusion of normal and polite forms of address, normal manners, formality  Exclusion of professionalism, objectivity | P24: ‘Because I come from a generation where that's the normal thing to do.’  P27: ‘It's just the right thing to do, it's polite.’ |
| Respect/appreciation | Expressions of respect and appreciation for the other person | Inclusion of respectful and appreciative use of language, dignity  Exclusion of recognition as an adult | P7: ‘I want to be seen as a dignified person.’  P17: ‘Because it's respectful.’  P34: ‘It makes me feel valued.’ |
| Individuality | Emphasis on personal perception, individuality of language, recognition as an adult | Inclusion of personal and individual address, perception as a person/patient  Exclusion of dignity | P10: ‘It's more personal for me.’  P25: ‘Because I think the nurse recognises me as a person and not as someone who is dependent.’ |
| Warmth/sympathy | Statements that mention warmth in language, a friendly tone or sympathy | Inclusion of warm, affectionate language, kindness, empathy/sympathy, trust | P2: ‘The nurse was lovely.’  P17: ‘It's more empathetic for me.’  P30: ‘I find it creates trust.’ |
| professional distance | Statements that emphasise professional address and maintaining distance | Inclusion of professionalism/level of language, distance/strangeness between patient and nurse, objectivity  Exclusion of formality | P8: ‘It's a professional form of address.’  P12: ‘There is still a certain distance between the patient and the nurse.’  P36: ‘It is a factual form of address.’ |
| age difference | Reference to age difference, generation-specific expectations | Inclusion of statements that emphasise the greater age of patients compared to nursing staff | P13: ‘I am at an age where I want to be addressed formally.’  P38: ‘It also depends on the age of the nurse. Younger nurses are not allowed to address me informally.’ |

**Category for preference of elderspeak:**

| **Category** | **Definition** | **Coding rule** | **Example quotations** |
| --- | --- | --- | --- |
| trust | Expression of trust | Inclusion of statements that emphasise trust and well-being | P15: ‘I feel comfortable there, I trust the nurse.’ |

**Categories for no preference of language style:**

| **Category** | **Definition** | **Coding rule** | **Example quotations** |
| --- | --- | --- | --- |
| Satisfaction with both language styles | Assessment or feeling of satisfaction regarding the use of both language styles | Inclusion of positive assessments of both language styles | P29: ‘I think everything is good.’  P37: ‘Because everything sounded nice.’ |
| Low relevance | Statements that assess the relevance of language style and personal significance as low | Inclusion of low significance/importance of language | P21: ‘I don't really care.’ |

Research question 2: What thoughts and feelings do patients have about the neutral language style and elderspeak?

**Categories for the neutral language style**

| **Category**  As a patient I feel… | **Definition** | **Coding rule** | **Example quotations** |
| --- | --- | --- | --- |
| well/good | Statements describing the language as normal and satisfactory | Inclusion of positive, neutral thoughts and feelings, thoughts on the topic of well-being, pleasantness | P2: ‘I feel good.’  P5: ‘I have a pleasant feeling about the language.’ |
| respected | Statements showing that patients feel respected and valued | Inclusion of concrete expressions of respect and appreciation | P16: ‘I would feel respected there.’  P39: ‘I feel valued.’ |
| treated personally | Reference to personal treatment, consideration of individual needs | Inclusion of personal recognition, perception as a human being | P13: ‘I would feel that I was being treated as an individual.’  P24: ‘I would feel that the nurse was responding to my needs.’ |
| cared for professionally | Statements emphasising professionalism and competence | Inclusion of objectivity and correct, professional and informative manners | P8: ‘As a patient, I feel that I am being treated correctly. The nurse did a professional job.’ |

**Categories for Elderspeak**

| **Category**  As a patient I feel… | **Definition** | **Coding rule** | **Example quotations** |
| --- | --- | --- | --- |
| infantilised/put down | Statements emphasising belittlement and childish treatment | Inclusion of feelings of infantilisation, patronisation, cognitive disregard, dehumanisation, unequal treatment and humiliation | P18: ‘I feel like I'm being treated like a child. I would feel unworthy.’  P32: ‘That makes me feel degraded.’  P44: ‘As if I were a bit stupid (...) as an old woman you can do anything to.’ |
| uncomfortable/not well | Statements expressing negative feelings without particular emotional colouring | Inclusion of references to negative thoughts and feelings such as not feeling well, dislike, perception as disturbing, unpleasant, strange, not right | P10: ‘I don't like that at all. It's not normal and I wouldn't find it attractive.’  P33: ‘That's not right.’ |
| treated impersonally | Statements describing impersonal and strange behaviour | Inclusion of feelings of impersonal or strange communication | P5: ‘That's impersonal.’  P4: ‘I feel strange.’ |
| neutral/no feelings | Statements without discernible feelings or emotional evaluation, neutral attitude | Inclusion of insensitivity, perception of no disturbance or discrimination | P29: ‘I don't develop any particular feelings about it.’  P31: ‘It wouldn't bother me if someone addressed me that way.’ |
| not respected | Statements that show a lack of respect or appropriate appreciation | Inclusion of feelings of disrespect | 17: ‘That's disrespectful, you don't do that.’ |
| positive perception | Statements expressing satisfaction and a positive assessment | Inclusion of positive thoughts/feelings | P15: ‘I feel flattered. I would be very happy if there were nurses like that.’ |

Research question 3: How do patients evaluate neutral and elderspeak language styles?

**Categories for neutral language style:**

| **Category**  *The way of speaking was...* | **Definition** | **Coding rule** | **Example quotations** |
| --- | --- | --- | --- |
| normal/good | Statements that evaluate the language style as normal and positive, without any particular emotional connotation | Inclusion of a positive evaluation such as normal, good, okay, showing satisfaction with the language style  Exclusion of specific evaluations | P35: ‘That's good, that's fine.’  P45: ‘I'm happy with that.’  P43: ‘It was alright.’ |
| warm/friendly | Reference to language that conveys warmth, friendliness and sympathy | Inclusion of friendliness, evaluation of manner of speaking as nice, reassuring, appropriate, natural, calm, endearing | P2: ‘The nurse was friendly.’  P22: ‘That was nice, it wasn't over the top.’  P12: ‘I think it was appropriate.’ |
| professional | Statements describing the language style as competent and professional | Inclusion of clear and distinct language that is understandable and factually correct | P4: ‘That was factual.’  P39: ‘That was clear and understandable.’  P8: ‘I think the way of speaking was professional.’ |
| respectful | Language that expresses respect, appreciation and appropriate politeness | Inclusion of a polite, respectful assessment of language style | P33: ‘That was respectful, like how you treat an adult.’  P16: ‘I think it was polite.’ |
| personal | Statements that emphasise a personal and needs-oriented manner of speaking | Inclusion of an assessment that refers to individual needs, perception as a human being | P2: ‘The nurse (...) responded to the patient's needs.’  P17: ‘The nurse perceived the person as a human being.’ |
| negative perception | Assessment of language as formal, rehearsed or superficial | Inclusion of a dismissive, fast and direct manner of speaking | P15: ‘I found the language dismissive and very fast.’  P26: ‘Seemed a bit rehearsed to me.’ |

**Categories for Elderspeak**

| **Category**  *The way of speaking was...* | **Definition** | **Coding rule** | **Example quotations** |
| --- | --- | --- | --- |
| childish/exaggerated | Statements describing Elderspeak as overly childish, exaggerated and dominant | Inclusion of an assessment as silly, exaggerated, childish, bossy, dominant, harsh | P26: ‘The nurse talks as if to a child.’  P42: ‘Imperious, as if you have to do what the nurse says.’  P22: ‘It's exaggerated because the way of speaking was somehow too cool, too familiar.’ |
| disrespectful | Statements describing elderspeak as disrespectful and demeaning | Inclusion of demeaning, disparaging and disrespectful language | P28: ‘As if the patient were not an equal, it is disrespectful.’  P18: ‘A bit demeaning.’ |
| cold/inappropriate | Statements describing the language as cold, inappropriate and emotionally distant | Inclusion of cold, unfriendly, unpleasant, condescending, derogatory and distant language | P24: ‘Was not appropriate.’  P3: ‘The language was too cold for me.’  P42: ‘Was condescending.’ |
| not good | Statements expressing dislike or dissatisfaction with elderspeak | Inclusion of dislike, disturbing perception | P2: ‘I didn't like it at all.’  P23: ‘Not particularly favourable. The basic principle of personal address should be different.’ |
| impersonal/too personal | Statements that rate Elderspeak as too distant or inappropriately personalised | Inclusion of ratings as impersonal or too personal, and failure to build trust or establish contact | P3: ‘Everything is too impersonal.’  P42: ‘It was too personal.’  P5: ‘Does not establish contact or trust.’ |
| positive perception | Statements that generally rate elderspeak as good or acceptable, without any particular emotional connotation | Inclusion of positive ratings | P31: ‘It's acceptable. It's a normal way of speaking. Not entirely commendable, but still acceptable.’ |

Research question 4: How do patients perceive the relationship between nurse and patient in the neutral and elderspeak videos?

**Categories for neutral speech**

| **Category**  The relationship between the nurse and the patient was... | **Definition** | **Coding rule** | **Example quotations** |
| --- | --- | --- | --- |
| normal/good | Interaction described as normal to positive without any particular emphasis on emotion | Inclusion of normal, satisfactory relationship  Exclusion: strong emotional connotation | P19: ‘Normal and everyday when you're in that situation as a patient.’  P43: ‘Just as it should be, normal.’ |
| professional | Statements describing the relationship as correct and professional | Inclusion of objectivity, formal distance, strangers, correctness | P33: ‘Was distant, which is how I would want to be treated.’  P4: ‘Was correct and objective.’ |
| respectful | Statements that describe the interaction as respectful and balanced | Inclusion of balance in the relationship, respect | P2: ‘The patient and nurse were on the same wavelength.’  P41: ‘Was respectful.’ |
| negative perception | Statements describing the relationship as brief, sterile and short on words | Inclusion of little communication on the part of the patient, sterile or no relationship, rapid conversation by the nurse | P20: ‘It was a bit sterile.’  P26: ‘There was no relationship between the two. The nurse did her job.’ |
| warm/friendly | Statements describing the relationship as friendly with positive connotations | Inclusion of warmth, friendliness, pleasant and trustworthy relationship | P42: ‘I would say it was pleasant.’  P16: ‘As a patient, you build up trust and feel safe.’ |
| personal | Statements that evaluate the relationship as personal with individual attention | Inclusion of personal needs, perception as a personality | P39: ‘The patient was recognised as a personality.’ |

**Categories for Elderspeak**

| **Category**  The relationship between the nurse and the patient was... | **Definition** | **Coding rule** | **Example quotations** |
| --- | --- | --- | --- |
| not good | Statements that evaluate the relationship negatively without a specific emotional attitude | Inclusion of no or poor relationship quality, dismissive relationship | P23: ‘There was no relationship between the patient and the nurse.’  P9: ‘It wasn't so good.’ |
| disrespectful/hierarchical | Statements emphasising dominance, hierarchy and disrespect in the relationship | Inclusion of disregard, disrespect, belittling of the patient through hierarchy, dominance of the nurse | P17: ‘The nurse has no compassion for the patient and felt superior to her.’  P28: ‘The relationship is as if the patient were the lowest of the low.’ |
| cold/inappropriate | Statements emphasising an emotionally distant, cold and strange relationship | Inclusion of cold language without compassion, intrusive and strange | P25: ‘Intrusive on the part of the nurse.’  P8: ‘A bit overbearing.’  P20: ‘“Strange”' |
| childish/too familiar | Statements emphasising the relationship as childlike and overly familiar, and an excessive degree of personal closeness | Inclusion of the childlike nature of the relationship, overly personal and familiar, buddy-like and too intimate | P22: ‘Too intimate, as if we've known each other forever.’  P41: ‘The relationship was as if the patient were a child.’  P42: ‘Very familial, which doesn't really belong there.’ |
| impersonal | Statements that miss a personal reference in the relationship | Inclusion of impersonal evaluation of the relationship | P33: ‘That was completely impersonal.’ |
| positive perception | Statements describing the relationship as normal to positive, but without strong emotionalisation | Inclusion of normal, good relationship assessment | P37: ‘I would say it was actually good.’  P29: ‘It was fine.’ |

# 7 Appendix 7: Test for normal distribution: Kolmogorov-Smirnov test and Shapiro-Wilk test

| **Items** | **Kolmogorov-Smirnov-test** | | **Shapiro-Wilk-test** | |
| --- | --- | --- | --- | --- |
|  | statistics | significance (*p*) | statistics | significance (*p*) |
| **Neutral Speech** | | | | |
| 1 | .509 | <.001 | .435 | <.001 |
| 2 | .479 | <.001 | .514 | <.001 |
| 3 | .360 | <.001 | .673 | <.001 |
| 4 | .329 | <.001 | .759 | <.001 |
| 5 | .411 | <.001 | .650 | <.001 |
| 6 | .520 | <.001 | .312 | <.001 |
| 7 | .500 | <.001 | .443 | <.001 |
| 8 | .241 | <.001 | .850 | <.001 |
| **Elderspeak** | | | | |
| 1 | .263 | <.001 | .781 | <.001 |
| 2 | .333 | <.001 | .715 | <.001 |
| 3 | .239 | <.001 | .814 | <.001 |
| 4 | .247 | <.001 | .859 | <.001 |
| 5 | .295 | <.001 | .770 | <.001 |
| 6 | .283 | <.001 | .777 | <.001 |
| 7 | .202 | <.001 | .891 | <.001 |
| 8 | .395 | <.001 | .682 | <.001 |
| Item 1: I feel that I am being treated disrespectfully as a patient.  Item 2: I found the language used to be appropriate and dignified.  Item 3: I feel that I can act independently and make my own decisions freely.  Item 4: I feel that I am dependent.  Item 5: I feel valued by the nursing staff.  Item 6: I feel that I am being talked down to as a patient.  Item 7: I notice that my self-confidence is decreasing.  Item 8: The way I was spoken to increased my self-esteem.  Likert scale: 1: strongly disagree. 2: somewhat disagree. 3: neutral. 4: somewhat agree. 5: strongly agree. | | | | |

# 8 Appendix 8: Results of the Bonferroni-Holm correction

| Item | Wilcoxon-Signed-Rank-test (*p*-Wert) | Threshold values of the Bonferroni-Holm correction |
| --- | --- | --- |
| 1 | < .001 | 0.006 |
| 2 | < .001 | 0.007 |
| 3 | < .001 | 0.008 |
| 4 | < .001 | 0.010 |
| 5 | < .001 | 0.013 |
| 6 | < .001 | 0.017 |
| 7 | < .001 | 0.025 |
| 8 | < .001 | 0.050 |

# 9 Appendix 9: Order of video presentation and effect on speech perception

To test whether the effect of speech condition (elderspeak vs. neutral) depended on presentation order, we fitted cumulative-link mixed models (CLMMs) for the ordered item responses (1–5) with a random intercept for participant. Prior to modelling four negatively worded items were reverse-scored so that higher values uniformly indicate a more positive perception; age was mean-centred and sex coded as a categorical covariate. The primary interaction model had the form

response_ij_∼condition_i_×order_vari_i_+age_c_i_+sexi+(1∣id_j_)

fitted with an ordinal logit link (adaptive Gauss–Hermite quadrature, nAGQ = 5). For interpretability and robustness we also fitted linear mixed models (LMMs) on the numeric 1–5 response with the analogous fixed-effect structure (response_num ~ condition * order_var + age_c + sex + (1 | id)). Evidence for an interaction was assessed by (a) inspection of the interaction coefficient in the CLMM and (b) a likelihood-ratio test comparing the additive covariate model (condition + order_var + age_c + sex + (1 | id)) to the interaction model. When appropriate, post-hoc estimated marginal means and pairwise contrasts (emmeans) were used to obtain cell-specific latent estimates and to compute odds ratios and predicted category probabilities (by combining model thresholds and cell-specific linear predictors). All analyses were conducted in R (packages: ordinal, lme4, emmeans, dplyr); models were run on the complete-case covariate dataset (554 observations, 45 participants). Statistical significance was evaluated at α = 0.05 and effect sizes and 95% confidence intervals are reported alongside p-values.

Interaction test. We tested whether the effect of condition (elderspeak vs. neutral) depended on presentation order (neutral first vs. ES first) by including the interaction condition × order_var in a cumulative-link mixed model (CLMM; logit link, random intercept for subject), adjusted for centered age and sex. The interaction term was not significant (condition × order_var: β = 0.045, SE = 0.346, z = 0.13, p = 0.898), and a likelihood-ratio test comparing the additive model to the interaction model was null (LR = 0.017, df = 1, p = 0.898). In the interaction CLMM the condition effect (ES vs. neutral) was negative but not significant (β = −0.327, SE = 0.243, z = −1.34, p = 0.179). Estimated marginal latent means (CLMM emmeans) were consistent across order levels: neutral_first — neutral = −1.22, ES = −1.54 (difference ≈ 0.32); ES_first — neutral = −1.19, ES = −1.47 (difference ≈ 0.28). Pairwise contrasts (neutral − ES) within order levels were directionally consistent (neutral_first: estimate = 0.327, SE = 0.243, p = 0.179; ES_first: estimate = 0.282, SE = 0.246, p = 0.251). Thus, there is no evidence that the elderspeak effect differs by viewing order; the elderspeak effect (neutral > ES) is of similar magnitude in both order groups but does not reach conventional significance on the ordinal scale.
